# INDUSTRY ISSUES: Pharmaceutical Factories as a Source of Drugs in Water

**DOI:** 10.1289/ehp.118-a383

**Published:** 2010-09

**Authors:** Rebecca Kessler

**Affiliations:** **Rebecca Kessler**, based in Providence, RI, writes about science and the environment for various publications. She is a member of the National Association of Science Writers and the Society of Environmental Journalists

Low levels of active pharmaceutical ingredients (APIs) turning up in natural waterways and drinking water supplies are coming under increasing scrutiny for their potential health effects on people and wildlife. Human waste has been identified as the main source of these pharmaceuticals, along with the common practice of flushing unused medications down the toilet. Now, a new study by the U.S. Geological Survey (USGS) highlights a largely overlooked contributor: pharmaceutical manufacturers.^1^ Effluent from two U.S. wastewater treatment plants that received discharge from pharmaceutical manufacturing facilities had levels of seven APIs that were 10–1,000 times higher than effluent from plants that received no such waste.

Between 2004 and 2009, USGS researchers sampled effluent and receiving water downstream from three wastewater treatment plants in New York State. Two of the plants received about 20% of their waste from pharmaceutical manufacturing facilities; the other received none. Researchers also collected effluent samples from 23 treatment plants around the nation that did not serve pharmaceutical manufacturers.

The researchers analyzed the samples for seven APIs (see box). In effluent from the two treatment plants serving pharmaceutical manufacturers, median concentrations of the most common APIs ranged from 2 to 400 μg/L. The researchers also found surprisingly high maximum concentrations of 1,700 μg/L for oxycodone and 3,800 μg/L for metaxalone. Moreover, low levels of two of the APIs turned up in a drinking water reservoir 30 km downstream from one plant. By contrast, in effluent from treatment plants with no pharmaceutical manufacturers among their clientele, concentrations of individual APIs rarely exceeded 1 μg/L, a figure that aligns with previous findings from treatment plant effluent in the United States and Europe.^2^,^3^

According to the authors, the USGS study is the first to directly link high concentrations of APIs in water to pharmaceutical manufacturers in the United States. This study follows a 2007 report of unprecedentedly high levels of API residues in effluent from an Indian treatment plant serving some 90 pharmaceutical manufacturers.^4^ The antibiotic ciprofloxacin occurred at levels up to 31,000 μg/L—more concentrated than the maximum therapeutic levels in human plasma.

Scientists have assumed those findings wouldn’t translate to the Western world, in part because the high market value of pharmaceutical products presumably motivates manufacturers to recover as much as possible and keep discharges to a minimum. “The conventional wisdom when the India paper came out was, ‘Well, that just couldn’t happen here.’ . . . And in fact, it did happen,” says Patrick J. Phillips, the present study’s lead author. He adds that it remains to be seen whether the API levels his team found apply to effluents from other U.S. pharmaceutical manufacturers.

The human health effects of waterborne APIs are largely unknown. Phillips points out that high concentrations of some APIs would raise greater concern than others, such as antibiotics that may promote drug resistance in bacteria. Of the seven drugs his team measured, five are federally controlled substances,^5^ and a quick calculation suggests that for oxycodone, for instance, a person would need to drink just 1.4 L of effluent containing the maximum concentration detected to ingest the lowest commercial dose of the drug.^6^

But of course, people usually do not drink effluent, and while praising the study, Christian Daughton, chief of the Environmental Chemistry Branch at the U.S. Environmental Protection Agency (EPA), points out that what really matters for human health are API levels in finished drinking water. So far these have typically been found only at minute nanogram-per-liter concentrations. “There’s very little evidence that just about any chemical at that level has an effect on humans,” Daughton says. Of greater concern is the potential effect on aquatic life, since even low levels of antidepressants and endocrine disruptors commonly found in sewage can profoundly affect fish and other organisms.^7^,^8^

The USGS findings also raise the question of transparency. U.S. manufacturers have no obligation to disclose what APIs they produce or discharge, so Phillips’s team relied on a time-consuming “forensic” approach: they chose the seven compounds for analysis only after noticing unusual chromatograph spikes in water samples, and then painstakingly developed detection methods for each one. Phillips says that since he’s shown that APIs from factories can get through treatment plants and into reservoirs, manufacturers should share more information with environmental monitors.

In response to the study, the Pharmaceutical Research and Manufacturers of America asserts that factories comply with environmental regulations and that APIs detected in surface waters “come primarily from patient use.” The pharmaceutical company Pfizer comments, “Previous studies have indicated . . . that the contribution from manufacturing operations is negligible. We look forward to subsequent work in this area to further understand the issue.”

Attention to the patient side of the equation has been gaining momentum. Take-back programs are cropping up across the nation as a way for consumers to safely dispose of unused medications.^8^ And the U.S. Senate’s Special Committee on Aging held a hearing on the subject in June. Committee chair Herb Kohl (D–WI) called for more take-back and waste-reduction programs to be implemented and for harmonization of contradictory federal guidelines on proper pharmaceutical disposal. Opening the hearing, he echoed the sentiments of many scientists when he stated, “While we don’t know yet what impact this has on humans, we can all agree that it’s disturbing to think about leftover drugs tainting our drinking water.”^10^

## Figures and Tables

**Figure:**
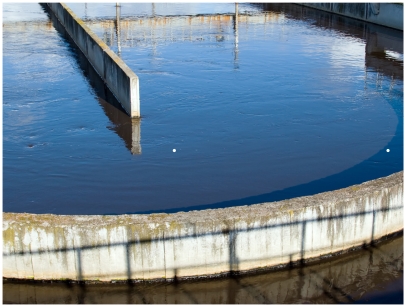
**Table:** 5 of the 7 drugs measured are federally controlled

Drug	Usage
methadone	opioid pain reliever
oxycodone	opioid pain reliever
butalbital	barbiturate
carisoprodol	muscle relaxant
metaxalone	muscle relaxant
diazepam	tranquilizer
phendimetrazine	amphetamine
